# Antioxidant Capacity and Cytotoxic Effects of Catechins and Resveratrol Oligomers Produced by Enzymatic Oxidation against T24 Human Urinary Bladder Cancer Cells

**DOI:** 10.3390/antiox8070214

**Published:** 2019-07-10

**Authors:** Claudia Lizet Meneses-Gutiérrez, Jacqueline Hernández-Damián, José Pedraza-Chaverri, Isabel Guerrero-Legarreta, Dario Iker Téllez, María Eugenia Jaramillo-Flores

**Affiliations:** 1 Departamento de Ingeniería Bioquímica, Escuela Nacional de Ciencias Biológicas, Instituto Politécnico Nacional, Wilfrido Massieu esq. Manuel Stampa s/n, Unidad Profesional Adolfo López Mateos, C.P. 07738 Mexico City, Mexico; 2 Departamento de Biología, Facultad de Química, Universidad Nacional Autónoma de México, Edificio F, Ciudad Universitaria, CP 04510 Mexico City, Mexico; 3 Biotecnología, Universidad Autónoma Metropolitana, Av. San Rafael Atlixco 186, Col. Vicentina, CP 09340 Mexico City, Mexico

**Keywords:** enzymatic polymerization, phenolic compounds, antioxidant activity, chelating capacity, bladder cancer

## Abstract

In this work the polymerization of catechin, epicatechin, and resveratrol was carried out through a peroxidase oxidation process in order to improve the biological activity of these phenolic compounds. The antioxidant activity of the oligomers was evaluated by their ability to scavenge reactive oxygen species (ROS) and their capacity to chelate metal ions Fe^2+^ and Cu^2+^. The antitumor effect of the oligomers was determined by their ability to induce toxicity in the T24 human bladder cancer cell line. By enzymatic peroxidase oxidation, it was possible to produce oligomers of catechin, epicatechin, and resveratrol with antioxidant capacity significantly higher than their preceding monomers. The ROS scavenging capacity of the oligomers was 20 times higher than that of the monomers, while the ability of the oligomers to chelate metal ions increased up to about 1000 times. Our data show the antitumor effect of the oligomers of catechin, epicatechin, and resveratrol in the T24 cell line, which was similar to that observed with cisplatin. Oligomers of catechin, epicatechin, and resveratrol have great potential to be used as therapeutic agents for the treatment of oxidative stress-related diseases and bladder cancer.

## 1. Introduction

In the last decade, the scientific and medical communities have performed a significant number of studies evaluating the biological properties of phenolic compounds (PCs) and their effects on human health [1,2,3,4]. It has been demonstrated, in vivo and in vitro, that catechin, epicatechin, and resveratrol, which can be found in cocoa, tea, and red wine, prevent and improve treatment of chronic degenerative diseases such as cancer [5,6], diabetes [7], and neurodegenerative [8] diseases, among others. The study of the mechanisms by which PCs are involved, in cancer and other diseases, is still insufficient. However, it has been observed that PCs have diverse effects on these diseases due to their antioxidant [9] and antimutagenic [10] properties.

The chemical structure of PCs is critical for their biological activity. It has been observed that PCs polymers found in food [11], and those obtained by chemical [12] or enzymatic [13] oxidation, show superior biological properties related to their respective preceding monomer. This explains why the process of enzymatic polymerization has been employed as a strategy to potentiate the biological activity of PCs. The search for new polymerization systems, as well as the optimization of the conditions for the polymerization process, is the subject of new investigations.

Cancer incidence worldwide is increasing every year, so there is a growing interest in the medical and scientific communities to find new anticancer agents from renewable sources, which are more efficient and less toxic. Bladder cancer has an important incidence in the United States of America: in 2018 there were approximately 80,470 new cases and 17,670 deaths [14]. In vivo studies have shown that PCs such as epigallocatechin gallate [2] and resveratrol [15] show anticancer activity on human bladder cancer T24 cells; however, hitherto there are no studies evaluating the anticancer effect of PCs oligomers on the T24 cell line. Therefore, the objective of this study was to obtain oligomers of catechin, epicatechin and resveratrol by enzymatic oxidation, free of toxic agents and without extensive and costly purification treatments, with antioxidant and anticancer properties superior to those of the corresponding preceding monomer.

## 2. Materials and Methods

### 2.1. Reagents

The enzyme used in the study was horseradish peroxidase type II (EC 1.11.1.7) and the substrates used were (+)-catechin hydrate, (–)-epicatechin, and resveratrol, of high performance liquid chromatography (HPLC) grade. Folin–Ciocalteu’s phenol reagent and sodium carbonate (Na_2_CO_3_) were used for the total phenolic content (TPC) determination. 2,2′-Azobis (2-methylpropionamidine) dihydrochloride (AAPH), fluorescein sodium salt, and Trolox were used for the oxygen radical absorbing capacity (ORAC) assay. Reagents for chelation activity assessment were ethylendiaminetetraacetic acid (EDTA), ferrous chloride tetrahydrate (FeCl_2_·4H_2_O), ferrozine, cupric sulfate pentahydrate (CuSO_4_·5H_2_O), and pyrocatechol violet (PV). Reagents for buffer preparation were sodium acetate, acetic acid, potassium phosphate monobasic anhydrous, and potassium hydroxide. The enzyme and reagents above mentioned as well as 3-(4,5-dimethylthiazol-2-yl)-2, 5- diphenyltetrazolium bromide (MTT) were purchased from Sigma-Aldrich (St. Louis, MO, USA). Dulbecco’s modified Eagle’s medium (DMEM) GIBCO BRL, TrypLE^TM^ Express GIBCO BRL were from Life Technologies Corporation (Carlsbad, CA, USA). Fetal bovine serum (FBS) and penicillin/streptomycin were from PAA Laboratories Inc. (Etobicoke, ON, Canada).

### 2.2. Synthesis of Oligomers of Catechin, Epicatechin and Resveratrol Through Enzymatic Oxidation

The enzymatic reaction (ER) of catechin, epicatechin or resveratrol was carried out using peroxidase and hydrogen peroxide (H_2_O_2_) in the dark in a shaking water bath PolyScience (Niles, IL, USA) at 100 cycles/min and 25 °C in a final reaction volume of 10 mL. Auto-oxidation of PCs was evaluated in the control reaction (CR), in which phosphate buffer with H_2_O_2_ substituted the solution of peroxidase. Peroxidase was dissolved in 0.1 M phosphate buffer (pH 6) and added to the reaction medium in order to obtain a final concentration of 2.5 units/mL. Stock solutions of the substrates at a concentration of 15 mM were prepared; catechin and epicatechin were dissolved in 30% ethanol, while resveratrol was dissolved in 50% ethanol, ensuring the solubility of the substrates in the reaction medium. Stock solutions were added to the reaction medium to reach a final substrate concentration of 1.5 mM. The oxidation reaction was started by adding three aliquots of 3% H_2_O_2_ at 0, 5, and 10 min, to obtain a final concentration of 0.3% H_2_O_2_ in the reaction medium. The ER was carried out for 24 h.

The reactions were monitored by analyzing the ultraviolet (UV)-visible (Vis) spectra, as well as by determining total phenolic content (TPC) and antioxidant capacity. For the latter, an aliquot of the reaction medium was taken and centrifuged at 4500 g for 5 min in a microcentrifuge (Sorvall Biofuge Primo R, Thermo Fisher Scientific, Erlangen, Germany), using the soluble phase for measurements.

#### 2.2.1. UV-Vis Absorption Spectra of Reaction Substrates and Products

The analysis of the oxidation of the PCs, as well as the evaluation of the formed products, was done by UV-Vis spectroscopy. The reaction medium was analyzed in the wavelength range of 190–1000 nm using a spectrophotometer UV-Vis 10S Genesys (Thermo Fisher Scientific, Madison, WI, USA). Analyses were performed at 10 min after the first aliquot of H_2_O_2_, then after every hour for 8 h and a final reading at 24 h.

#### 2.2.2. Determination of the Consumption of Substrate and Formation of Product through Reversed Phase (RP)-HPLC

The evaluations of substrate consumption and product formation in the CR and ER were performed by reversed phase high performance liquid chromatography (RP-HPLC) [16]. The analysis was carried out with an HPLC Agilent 1260 Infinity (Santa Clara, CA, USA) with a quaternary pump and diode array detector, and a C18 Zorbax SB-C18 5-µm column, measuring 4.6 cm × 150 mm. Data were processed with OpenLAB CDS software. The mobile phases employed were 2.5% acetic acid (A) and acetonitrile (B) (HPLC grade from Sigma-Aldrich) with an elution gradient from 0 to 10% B for 5 min, from 10 to 30% B for 20 min, and from 30 to 50% B for 20 min, flow 1 mL/min. In total, 20 µL of sample were injected, previously filtered through poly(vinylidene fluoride) (PVDF) syringe filters of 0.45 µm (Millipore, Burlington, MA, USA). Determinations were performed 10 min after the first aliquot of H_2_O_2_, then after every hour for 8 h and finally at 24 h. The detection of monomers and oligomers of catechin, epicatechin and resveratrol was performed at 250 and 280 nm.

#### 2.2.3. Characterization of Oligomers by Mass Spectrometry

The molecular weight and molecular weight profile of the reaction products were determined by Matrix-Assisted Laser Desorption/Ionization Mass Spectrometry (MALDI-TOF MS) using a mass spectrometer BrukerAutoflex (BrukerDaltonics, Billerica, MA, USA). Spectra were obtained on a matrix-assisted laser desorption/ionization spectrometer with a time-of-flight detector AutoFlex Speed. The samples were prepared by mixing 1.5 mL of the reaction with 1.5 mL of the matrix (2,5-dihydroxybenzoic acid, DHB). MALDI conditions were varied to obtain an appropriate spectrum adjusting the laser power (30–60%), with a voltage gain with ranges of 12–60 X for the reflectron phase and 10–300 ns for the ion extraction pulse. The interval for analysis was from 200 to 3500 *m/z*.

#### 2.2.4. Evaluation of the Total Phenolic Content (TPC) in the Reaction Solutions

The TPC in CR and ER was determined by the Folin–Ciocalteu method [17]. Samples from the CR and ER were taken at: 10 min, each hour from 1 h to 8 h, and 24 h. Samples of 100 µL of the supernatant were taken and mixed with 750 µL of a 1:10 dilution of Folin–Ciocalteu reagent in water; the mixture was incubated for 5 min and protected from light at room temperature. Then 750 µL of 6% Na_2_CO_3_ were added to the mixture and, after 90 min of incubation, the absorbance at 725 nm was determined. The reaction blank was prepared by using water instead of the supernatant. A standard curve was prepared and the TPC was expressed as µM catechin equivalents (CE). The concentration of the control and enzymatic reactions used in the methods of the following sections were estimated by this procedure.

#### 2.2.5. Determination of the Antioxidant Capacity by the ORAC Method

The principle of this method is based on the ability of antioxidant compounds to inhibit the loss in fluorescence of fluorescein induced by the AAPH radical. The antioxidant capacity is evaluated by calculating the area under the curve (AUC) in the graph of fluorescence intensity versus time. The measurement of fluorescence intensity of fluorescein was performed in a Synergy 2 microplate reader (Biotek Instruments Inc., Winooski, VT, USA) at an excitation wavelength of 480 nm and emission wavelength of 520 nm. Into the wells of a black flat bottom microplate, 20 µL of the supernatant of the reaction medium and 120 µL of fluorescein solution (120 nM) were placed. This microplate was incubated at 37 °C for 15 min and stirred for 2 min. Subsequently, 60 µL of 40 mM AAPH solution were added to the wells of the microplate. Measurements were made every min for 80 min, shaking the microplate for 5 s before each reading [18]. A calibration 1 curve of Trolox concentration against calculated AUC was plotted. AUC values of the sample were substituted in the equation of linear regression obtained from the calibration curve in order to express the results as µM Trolox equivalents (TE). The AUC was calculated by the following equation:

$$AUC=1+\sum_{t=0}^{t=80}\;\left(\frac{f_{1}}{f_{0}}\right)$$

where *F* is the ratio of the fluorescence of the blank at time 0, *f*_1_ is the fluorescence of the sample at each time, and *f*_0_ is the fluorescence of the blank at time 0. Blanks were prepared with the solvent containing the respective sample.

#### 2.2.6. Evaluation of the Fe^2+^ and Cu^2+^ ions’ Chelating Capacity

The methods used to determine the chelating ability of PCs and their oligomers are based on the quantification of the ferrozine–Fe^2+^ and PV–Cu^2+^ complexes. PCs and their oligomers chelate Fe^2+^ and Cu^2+^ ions from the reaction medium, inhibiting the formation of complexes [19]. For the analysis Fe^2+^ chelation, portions of 0.5 mL of sample were used, to which 2 mL of 0.1 M sodium acetate buffer (pH 4.9) and 50 µL of 2 mM FeCl_2_ were added; the mixture was incubated at 25 °C for 30 min. Then, 0.2 mL of 5 mM ferrozine were added. Incubation was then extended for 30 min and the absorbance of the solution was read at 562 nm. Analysis of Cu^2+^ chelation was done with 0.5 mL of sample, 2 mL of 50 mM sodium acetate buffer (pH 6) and 50 µL of 5 mM CuSO_4_, the mixture was incubated at room temperature for 30 min. Afterwards, 50 µL of 4 mM PV were added; the mixture was incubated for 30 min and the absorbance of the solution was read at 632 nm. Chelation ratios were calculated by the equation:

% Chelation = (A0 −A1 )/A0 × 100

where A0 is the absorbance of the negative control and A1 is the absorbance of the sample. Negative controls were prepared using water instead of the sample. First, the chelating capacity of the reaction media (CR and ER) was evaluated as a function of time. Thus, samples were taken at 10 min, and at every hour from 1 h up to 8 h, and at 24 h after the reaction started. Next, the concentration of sample required to chelate 50% of metal ions (IC_50_) was determined.

### 2.3. Evaluation of Cytotoxic Effects of Oligomers of Catechin, Epicatechin and Resveratrol in a Human Urinary Bladder Cancer Cell Culture

#### 2.3.1. Cell Culture

The T24 cell line (HTB-4^TM^) is derived from an invasive transitional cell carcinoma of human urinary bladder and was obtained from American Type Culture Collection (ATCC, Manassas, VA, USA). T24 cells were cultured in Dulbecco´s Modifies Eagle Medium (DMEM) (high glucose concentration) supplemented with 10% Fetal Bovine Serum (FBS), 0.33% sodium bicarbonate, and antibiotics (100 units/mL penicillin, 0.1 mg/mL streptomycin) under a humidified atmosphere of 5% CO_2_ at 37 °C.

#### 2.3.2. Cell Viability Assessed by MTT Reduction

T24 cells were treated with different concentrations (0.9, 4.5 and 9 µM CE) of the control and ER of catechin, epicatechin and resveratrol, for 24 h. Dimethyl sulfoxide was added simultaneously with the reaction products at a final concentration of 2%. In order to assess the changes in the cell density of the cells after treatment, photographs were taken with a phase contrast microscope inverted at 10× magnification (Nikon Eclipse TS100F, Nikon Co., Tokyo, Japan). After the different treatments, cells were incubated in medium containing MTT (0.125 mg/mL) for 1 h at 37 °C. Medium was discarded, and the purple colored precipitates of formazan crystals were dissolved in 800 µL of acidified isopropanol (0.04 N HCl). Absorbance was measured at 570 nm using a Synergy HT multi-mode microplate reader [20] (Biotek Instruments Inc., Winooski, VT, USA). The reduction in viability of T24 cells treated with the reaction products was expressed as percentage compared to non-treated cells. Control cells were considered to be 100% viable. Each MTT assay was repeated three times, using three wells per experimental condition.

### 2.4. Statistical Analysis

The experimental analyses were performed in triplicate and results are presented as means ± standard error of the mean (SEM). Excel 2007 and GraphPad, Prism version 5.00 were used for the graphical and statistical evaluations in this study. Statistical analysis was performed by one-way analysis of variance (ANOVA) followed by Tukey’s or Dunnett’s multiple comparisons tests. A *p*-value less than or equal to 0.05 was considered as statistically significant.

## 3. Results

### 3.1. Enzymatic Oxidation of Catechin, Epicatechin, and Resveratrol

**Figure 1:**
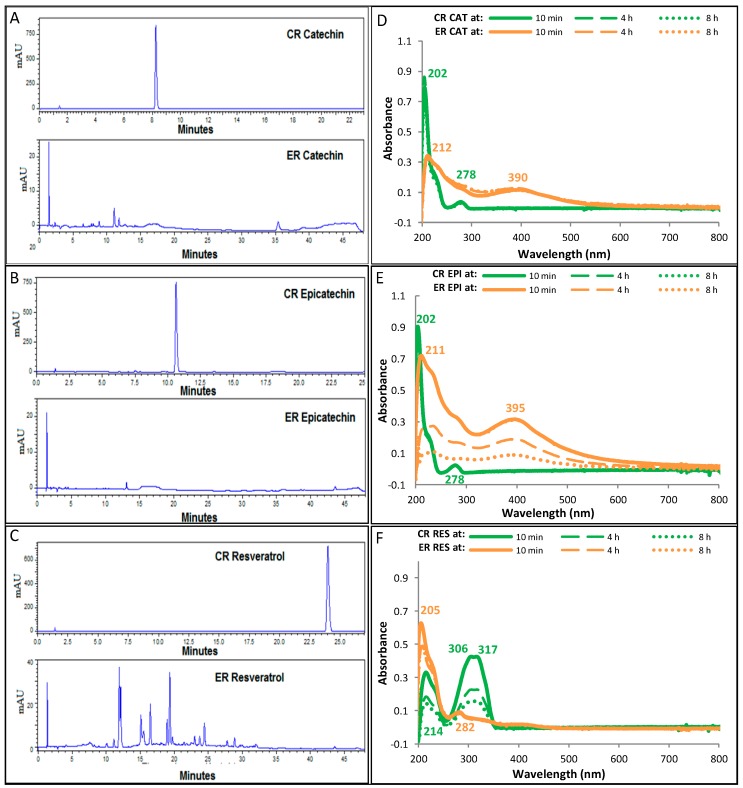
Reversed phase high performance liquid chromatography (RP-HPLC) chromatograms of the control reaction (CR) and enzymatic reaction (ER) with (**A**) catechin (CAT), (**B**) epicatechin (EPI), and (**C**) resveratrol (RES), at 10 min of reaction. The graphs (**D**–**F**) show the ultraviolet-visible (UV-Vis) absorption spectra of the CR and ER with each polyphenolic compound at different reaction times. Concentrations of phenolic compounds (PCs), peroxidase, and hydrogen peroxide (H_2_O_2_) in the reaction medium of the ER were 1.5 mM, 2.5 U/mL, and 0.05%, respectively. In the CR, peroxidase was substituted by phosphate buffer solution. mAU: milli arbitrary area units.

The enzymatic oxidation of catechin and epicatechin occurred since the first addition of H_2_O_2_ to the reaction medium, showing a drastic change in color due to the formation of dark compounds, as well as the generation of a precipitate. Figure 1A,B show that in the ER, catechin (retention time = 8.3 min) and epicatechin (retention time = 10.6 min) are consumed almost entirely after 10 min of starting the reaction. The products of the ER exhibit maximum absorption peaks at 212 and 390 nm for catechin (Figure 1D) and 211 and 395 nm for epicatechin (Figure 1E).

In the CR of catechin and epicatechin, neither changes in color nor precipitate formation were observed, suggesting that PCs were not oxidized by H_2_O_2_, nor due to autoxidation processes. In the UV-Vis absorption spectra of the CR obtained at different reaction times, it was found that catechin (Figure 1D) and epicatechin (Figure 1E) were not oxidized, as the peaks of maximum absorption were maintained at 202 and 278 nm, which are characteristic of catechin and epicatechin. In RP-HPLC chromatograms of the CR of catechin and epicatechin obtained after 24 h of reaction (data not shown), no significant differences were observed in the concentration of catechin and epicatechin with respect to the start of the reaction.

In the CR of resveratrol, turbidity and formation of a precipitate were observed in the solution from the first minutes of the reaction. However, the development of oxidation processes was discarded, since even after 8 h of reaction (Figure 1F) the wavelengths of maximum absorption, characteristic of resveratrol (214, 306, and 317 nm) remained unchanged, so that the turbidity and precipitation changes might be ascribed due to changes in the solubility of resveratrol. The chromatogram of RP-HPLC of Figure 1C shows that in the ER, resveratrol (retention time = 24 min) is rapidly oxidized, producing oxidation products with wavelengths of maximum absorption at 205 and 282 nm.

The peaks observed in the RP-HPLC chromatograms of the ER (Figure 1A–C), particularly in the ER with resveratrol, correspond to the formed products in the reaction media. In order to identify and quantified the products of the enzymatic reactions, purification procedures will to be done in the next stage of this work. However, at the moment, the obtained results are evidence that the established conditions in the enzymatic reactions are adequate to carry out the enzymatic oxidation process.

### 3.2. Evaluation of Molecular Weight Profile in the ER

By means of the analysis of the molecular weight of the reaction products through MALDI-TOF-MS, it was confirmed that in the CR, PCs did not undergo oxidation, so that in the reaction medium there were only monomers of catechin, epicatechin, and resveratrol. In the ER, a mixture of oxidation products was found, which consisted of two or more monomer units of catechin, epicatechin, or resveratrol.

**Figure 2:**
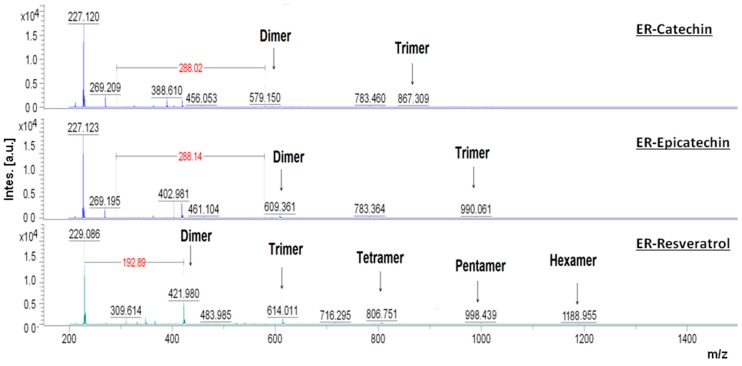
Molecular weight distribution in the enzymatic reaction (ER) with catechin, epicatechin and resveratrol. Mass spectra obtained through Matrix-Assisted Laser Desorption/Ionization Mass Spectrometry (MALDI-TOF-MS) with amplification from 150 to 1500 *m/z*. The samples were obtained from the ER medium after 30 min from the start of the reaction. The concentrations of phenolic compounds (PCs), peroxidase, and hydrogen peroxide (H_2_O_2_) in the reaction medium of the ER were 1.5 mM, 2.5 U/mL, and 0.05%, respectively.

In the mass spectra obtained by MALDI-TOF-MS presented in Figure 2 it is possible to observe that after 30 min of reaction between peroxidase and catechin, epicatechin and resveratrol, oligomers with different chain lengths were generated. In the ER with catechin, the ions found had *m/z* values of 579.15 and 867.306, that correspond to the formation of dimers and trimers. As with catechin, the ER with epicatechin produces dimers and trimers. Nonetheless, the mass of the ions was different (609.36 and 990.01), indicating that the structures of the formed oligomers were different. In the ER with resveratrol, the formation of resveratrol oligomers of up to six units were observed, with *m/z* values of 421.98, 614.01, 806.75, 998.44, and 1188.95.

### 3.3. Capacity of the Oligomers of Catechin, Epicatechin and Resveratrol to Scavenge Reactive Oxygen Species (ROS)

**Figure 3:**
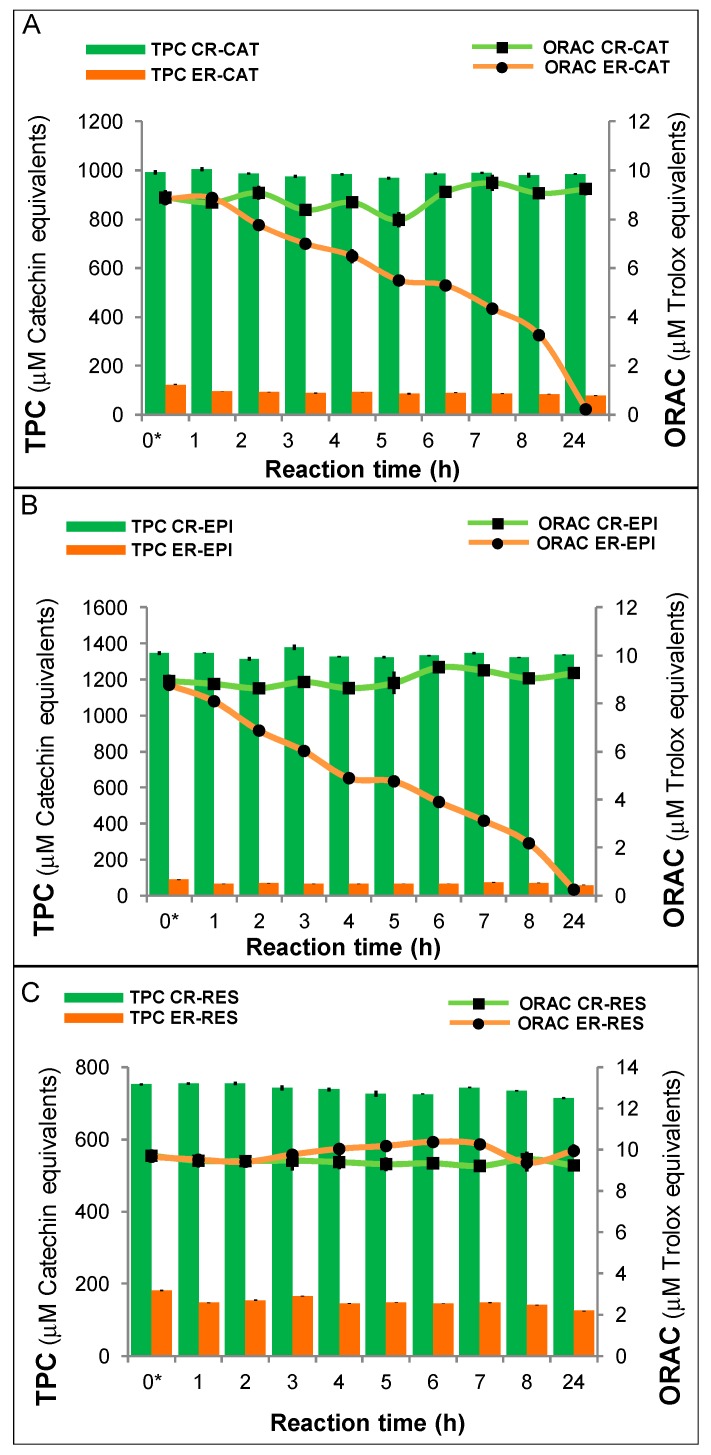
Relationship of the antioxidant capacity, determined by the oxygen radical absorbing capacity (ORAC) method, and the total phenolic content (TPC) in the reaction with (**A**) catechin (CAT), (**B**) epicatechin (EPI), and (**C**) resveratrol (RES) evaluated at different reaction times. Values are mean ± SEM, *n* = 3. Concentrations of phenolic compounds (PCs), peroxidase, and hydrogen peroxide (H_2_O_2_) in the reaction medium of the enzymatic reaction (ER) were 1.5 mM, 2.5 U/mL, and 0.05%, respectively. In the control reaction (CR), peroxidase was substituted by phosphate buffer solution.

The TPC in the control reactions (CRs) with catechin, epicatechin and resveratrol did not show significant differences throughout the reaction time (Figure 3A–C, clear bars), while the TPC, in the enzymatic reactions (ERs) (Figure 3A–C, dark bars), decreased drastically from the beginning of the reaction with respect to CRs. Considering the TPC values in the CRs at time 0 and those in the ERs at 24 h, it is possible to observe a decrease of 92%, 96%, and 98% in the TPC, in the reactions with catechin, epicatechin and resveratrol, respectively. The antioxidant capacity of the CRs with catechin, epicatechin, and resveratrol (Figure 3A–C, lines with squared markers), assessed by the ORAC method, did not show significant difference throughout the reaction. The antioxidant capacity in the ERs (Figure 3A–C, lines with circle markers) decreased gradually with progress of the reaction. However, considering the TPC of the CRs and ERs, it is noted that the antioxidant capacity of the products of the ERs is higher than that observed in the CRs. For example, after 1 h of reaction, the TPC of the CR of catechin was 1005.3 ± 8.3 μM CE, while the TPC of the ER significantly decreased up to 95.1 ± 0.4 μM CE. Nevertheless, no significant differences in antioxidant capacity were observed, since 8.7 ± 0.1 μM TE was the result with monomers (CR) and 8.9 ± 0.1 μM TE was obtained with oligomers (ER). Hence, the antioxidant capacity of the oligomers was about ten times higher than that of the monomers. In the reactions with epicatechin, the effect of polymerization on the antioxidant capacity was higher, since after 1 h of reaction, the TPC of the CR was 1348.2 ± 2.5 μM CE, while the TPC of the ER significantly decreased to 65.8 ± 0.7 μM CE, yielding 8.8 ± 0.2 μM TE with the monomers (CR) and 8.1 ± 0.2 μM TE with the oligomers. So, the antioxidant capacity of the epicatechin oligomers was about 20 times higher than that of the monomers. In the CR and ER of resveratrol, no significant differences were observed in the antioxidant capacity; however, significant differences were observed in the TPC obtained throughout the corresponding reactions. The antioxidant capacity of the oligomers of resveratrol was approximately five times higher than the antioxidant capacity of resveratrol monomers.

### 3.4. Chelating Capacity of Oligomers of Catechin, Epicatechin, and Resveratrol

Table 1 shows that the capacity of chelation of ions Fe^2+^ and Cu^2+^ of the oligomers of catechin, epicatechin and resveratrol produced in the ER was significantly higher than that observed in the monomers of the CR. The IC_50_ values of Fe^2+^ and Cu^2+^ ions were significantly lower than the IC_50_ of the monomers of catechin, epicatechin and resveratrol.

### 3.5. Anti-Viability Activity of the Oligomers of Catechin, Epicatechin and Resveratrol

**Figure 4:**
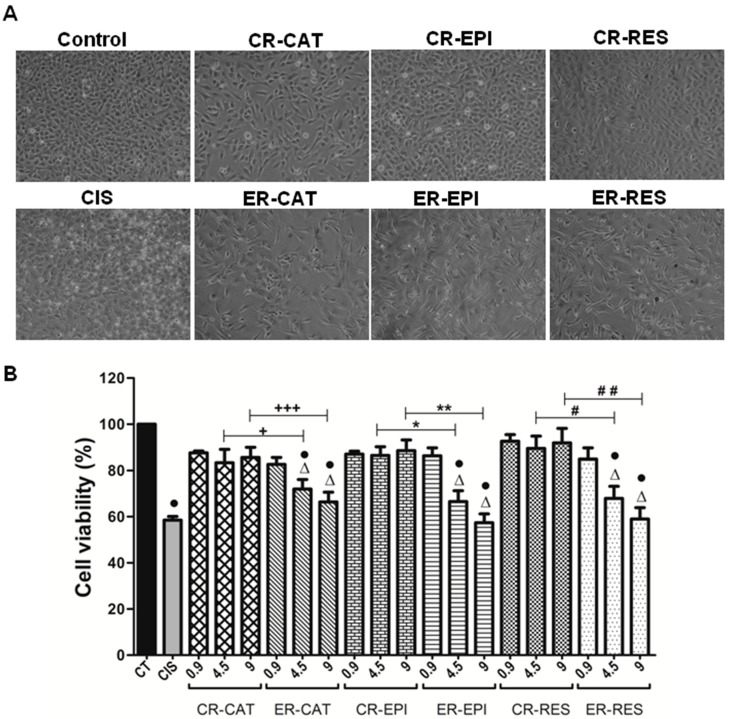
Cell damage in T24 bladder cancer cells induced by cisplatin and the products of the control (CR) and enzymatic (ER) reaction of catechin (CAT), epicatechin (EPI), and resveratrol (RES). (**A**) Representative images (10×) of the T24 cells’ density obtained by phase contrast microscopy and (**B**) cell viability of T24 cells assessed by the 3-(4,5-dimethylthiazol-2-yl)-2, 5-diphenyltetrazolium bromide (MTT) assay after different treatments after 24 h. T24 cells were treated for 24 h with cisplatin (10 mM CIS) or with different concentrations (0.9, 4.5 and 9 μM CE) of the CR and enzymatic ER of CAT, EPI, and RES. Control (CT) cells were treated for 24 h with 2% dimethyl sulfoxide (DMSO). Data are mean ± SEM, *n* = 3. • *p* < 0.05 vs. CT, + *p* < 0.0229 CR-CAT vs. ER-CAT (4.5 μM), +++ *p* < 0.0001 CR-CAT vs. ER-CAT (9 μM), * *p* < 0.0381 CR-EPI vs. ER-EPI (4.5 μM), ** *p* < 0.0018 CR-EPI vs. ER-EPI (9 μM), # *p* < 0.0144 CR-RES vs. ER-RES (4.5 μM), ## *p* < 0.0076 CR-RES vs. ER-RES (9 μM). ∆ Non-significant difference at *p* < 0.05 ER-CAT, ER-EPI, and ER-RES vs. CIS.

The oligomers of catechin, epicatechin and resveratrol showed a marked anti-viability effect on the T24 cell line of human urinary bladder transitional cell carcinoma. As depicted in Figure 4A, a noticeable decrease in cell density was observed when the cells were treated with oligomers, which is an outcome of their cytotoxic effects. The cytotoxic effects of the oligomers of catechin, epicatechin, and resveratrol depended on the concentration employed; thus, the highest cytotoxic effect was obtained with the oligomer concentration of 9 µM CE, reducing the viability of T24 cells after 24 h of treatment at 66.4%, 57.4%, and 59%, respectively.

Figure 4B shows that unlike the oligomers, monomers of catechin, epicatechin, and resveratrol did not exhibit antitumor effects on T24 cells, since after 24 h treatment with the monomers, cell viability did not decrease significantly with respect to the control. Figure 4B also shows that there were significant differences in the cytotoxic effect of monomers versus oligomers of catechin, epicatechin, and resveratrol at 4.5 and 9 µM CE concentration.

In order to evaluate the potential of the oligomers of catechin, epicatechin and resveratrol as chemotherapeutic agents, cisplatin was included in this study, which is an antineoplastic agent used in the treatment of different types of cancer. Figure 4A shows no significant differences between the viability of T24 cells treated with the oligomers of catechin, epicatechin, and resveratrol (4.5 and 9 µM) and T24 cells treated with cisplatin (10 µM).

## 4. Discussion

Epicatechin and catechin are good substrates for peroxidase, with the hydroxyl group of ring C being the preferred site for developing oxidation [21]. Both are diasteroisomers, which suggests that oxidation is carried out by the same mechanism, generating products with similar structures; this being the reason for the great similarity in maximum absorption wavelengths.

In the next stage of this work, purification procedures needed be carried out in order to determine the chemical structure of the oligomers produced in the ER. However, it is possible to assume that the chemical structures of the dimers and trimers of catechin are different to the dimers and trimers of epicatechin since their *m/z* values are different. By mass spectroscopy, three different dimers of catechin (*m/z* 579.15, 579.15, and 611.14) were formed in the reaction with peroxidase, two of them are structural isomers that have the same *m/z* value (579.15) than the dimer of catechin obtained in the present study. Such isomers differ only in the site where catechin radicals are joined to form dimers [22]. The phenoxy radicals tend to stabilize through the delocalization of their electric charge by the phenolic rings and -C=C- bonds [23], so that the union of two phenoxy radicals to form a dimer provides the possibility of more than two structures. The *m/z* values of the oligomers of resveratrol obtained in the ER showed that oligomers were produced by the subsequent union of units of *m/z* 190–192. This result suggests that the mechanism by which resveratrol is oxidized to form phenoxy radicals is consistent, indicating that there are preferable positions in the resveratrol’s structure susceptible to oxidation. The reaction of peroxidase and H_2_O_2_ oxidized PCs, generating phenoxy radicals that could join to each other by phenolic oxidative coupling reactions in order to form dimers, by covalent bonds of type C-C or C-O. The dimers formed in the oxidation reaction were novel peroxidase substrates, generating radicals of dimers that were joined together or with phenoxy radicals to form tetramers or trimers, respectively [24]. Therefore, at the end of the oxidation-polymerization reaction, oligomers and polymers were obtained with a dispersion of molecular weights.

Reduction of TPC in the ERs occurs because the phenolic compounds are rapidly oxidized by peroxidase and because some of the products precipitate in the reaction.

The PCs have the ability to scavenge free radicals, such as ROS, by donating hydrogen atoms and electrons [25]. PCs exhibit a favorable chemical structure for trapping free radicals, since it could be quickly stabilized by resonance. The number of hydroxyl groups and the position of these in the phenolic rings, determine the antioxidant potential of the PCs [26]. The structure of PCs oligomers formed through the enzymatic process will have a greater number of hydroxyl groups than the monomers, so that the antioxidant capacity will increase. However, if the polymer has a large number of monomer units, the polymer interaction with free radicals could be compromised, reducing their antioxidant capacity. Because of that, the gradual decrease of the antioxidant capacity of the oligomers of catechin and epicatechin, observed in this study, could be related to the steric hindrance generated in products with a high number of monomer units.

In some studies, it has been found that enzymatic polymerization processes allow to improve the biological properties of the PCs, such as their antioxidant capacity. However, the success of the improvement of the antioxidant capacity of the PCs depends on several factors such as: the polymerization process conditions, the oxidative potential of the enzymes used and the chemical nature of the PCs. The evaluation of the antioxidant capacity of PCs oligomers can be accomplished by different methods, so a correct comparison of the results obtained by different authors should be done. The lipid peroxidation capacity of PCs oligomers was four times higher than that observed with the monomer of catechin [22]. Polymers of polyesculin and polyrutin have superior capacity to inhibit the xanthine oxidase and chelation capacity compared to their respective monomers. However, it was also observed that the polymerization of rutin does not increase their free radical scavenging capacity [27]. Also, resveratrol has a greater capacity of 2,2-diphenyl-1-picrylhydrazyl (DPPH) radical scavenging than resveratrol dimer [28].

In previous studies it has been possible to obtain polymers of catechin [29], epicatechin [30] and resveratrol [31] by enzymatic oxidation with peroxidase; however, unlike those studies, in the present work a strictly aqueous reaction medium was used without the need to use toxic solvents such as acetone and methanol. Furthermore, in this work, polymers with increased antioxidant capacity were produced from 10 min of reaction, while in other studies oligomers were generated at between 2 and 24 h. The method used in this work is, therefore, more efficient at producing oligomers of catechin, epicatechin, and resveratrol.

The IC_50_ of the oligomers obtained in the ER is even lower than that obtained with EDTA, which is a widely recognized chelating agent. Possibly, the conformation and arrangement of the structures formed during the polymerization process increased the sites for interaction with metal ions, which promoted the chelating activity. The structure of PCs is decisive for their chelating capacity, since, as observed, PCs with catechol or galloyl groups have greater chelating ability than PCs not containing catechol or galloyl groups [32].

The presence of transition metals such as iron and copper in the human body is required for the development of the metabolic functions. However, since iron and copper generate reactive species and are joined by non-specific interactions to biomolecules, they could be a risk factor for developing diseases [33]. Iron accumulation in the human brain is related to the development of neurodegenerative chronic diseases such as multiple sclerosis, Parkinson’s disease, and Alzheimer’s disease. In vivo and in vitro studies have shown that due to their chelating and antioxidant properties, resveratrol [34] and catechins present in green tea [35] inhibit the processes involved in the development of diseases such as Parkinson’s disease and Alzheimer’s disease.

EDTA is a chelating agent used in chelation therapy to treat severe cases of metal poisoning. Chelation therapy has also been used in the treatment of atherosclerotic disease, where it has been employed to decrease the risk of cardiovascular events, primarily in diabetic patients [36]. In vivo and in vitro chelation therapy studies have shown antitumor effects in various cancers, as chelating agents diminish iron in neoplastic cells needed for replication [37].

The results of the present study are of great importance because this is the first work that shows a marked increase of the chelating ability of phenolic compounds by means of enzymatic polymerization. It is necessary to deepen the study of the chelating properties of the oligomers of catechin, epicatechin and resveratrol given that they have great potential as therapeutic agents that could be used in the treatment of diseases related to heavy metals toxicity, as well as antitumor activity on the cell line T24.

Although there are few studies on the evaluation of anticancer properties of the oligomers of PCs [38], it has been observed that the anticancer activity of the oligomers of PCs is higher than that of their corresponding monomers. However, there is no direct correlation between the number of monomer units and the extent of anticancer activity. The cytotoxic effect of vaticanol C (tetramer of resveratrol) on the colon cancer cell line (SW480) was about three times higher than that of veteriaphenol-A (octamer of resveratrol) and seven times that of monomeric resveratrol [39]. In the present study, the anticancer activity was attributed to a mixture of oligomers formed by different monomer units and not to a specific oligomer.

Cisplatin promotes oxidative stress in the organism due to the overproduction of ROS and inactivation of oxidative enzymes, causing nephrotoxicity, among other disorders [40]. The oligomers of catechin, epicatechin and resveratrol could be used as adjuvants in the treatment of bladder cancer with cisplatin, given that these oligomers might act as chemotherapeutic and antioxidant agents; while avoiding the oxidative damage caused by ROS. In general, PCs with large structures showed low bioavailability. Thus, for clinical studies, in vivo and in humans, these compounds should be administered intravesically, in order to ensure their action in the precise location where cancer has developed.

The study of the mechanisms involved in the antitumor effects of the oligomers of catechin, epicatechin, and resveratrol in the cell line T24 is an unexplored field for further research. In other cell lines it has been observed that the monomers and oligomers of resveratrol induced apoptotic processes by interrupting the cell cycle and by caspase activation, among other mechanisms, depending on the cellular microenvironment [41].

The matrix metalloproteinases (MMPs) are endopeptidases involved in cancer development, intervening in processes of angiogenesis, cell differentiation, proliferation, apoptosis, tumorigenesis and metastasis. Inactivation of MMPs is therefore one of the objectives sought for cancer therapies. The epigallocatechin gallate present in green tea inhibits the expression of MMP in the cell line T24 suppressing invasion and metastasis [42]. MMP contain Zn^2+^ in the active site, so that oligomers of catechin, epicatechin, and resveratrol could be used as chelating agents to inactivate MMP and stop the processes of cancer development.

## 5. Conclusions

The conditions employed in the enzymatic oxidation reaction were successful in producing oligomers of catechin, epicatechin, and resveratrol with biological activities higher than their respective monomers. The oxidation reaction with peroxidase produced oligomers of PCs after the first 10 min of reaction without requiring toxic solvents. The ability of catechin, epicatechin, and resveratrol to scavenge ROS considerably increased when they were polymerized. This is the first study reporting that the chelating capacity of the oligomers of catechin, epicatechin, and resveratrol was considerably higher than that of the precursor monomers and even superior to that of EDTA. In addition, the antiproliferative effect of the oligomers of catechin, epicatechin, and resveratrol on the T24 cell line of human bladder cancer showed a similar effect to that obtained with cisplatin. Due to their excellent antioxidant and anticancer properties, the oligomers of catechin, epicatechin, and resveratrol are an attractive possibility for new therapeutic agents from new sources.

## Figures and Tables

**Table 1:** IC_50_ values of monomers and oligomers of catechin, epicatechin, and resveratrol, obtained from the control and enzymatic reactions after 2 h of reaction.

Products and Standard.	Fe^2+^ Chelating Activity IC_50_ * (μM CE)	Cu^2+^ Chelating Activity IC_50_ (μM CE)
Control reaction:		
Catechin	1484.3 ± 3.2 ^b^	889.8 ± 7.2 ^d^
Epicatechin	1633.9 ± 32.5 ^a^	1105.8 ± 36.9 ^c^
Resveratrol	1484.4 ± 42.9 ^b^	937.1 ± 25.8 ^d^
Enzymatic reaction:		
Catechin	0.4 ± 0.02 ^g^	1.8 ± 0.02 ^g^
Epicatechin	0.5 ± 0.00 ^g^	1.7 ± 0.06 ^g^
Resveratrol	0.5 ± 0.02 ^g^	20.9 ± 0.3 ^g^
Standard:		
EDTA	125.2 ± 1.4 ^e^ (μM EDTA)	367 ± 6.1 ^f^ (μM EDTA)

Values are mean ± SEM, *n* = 3. No significant differences in the values that share the same letter. * IC_50_ is the concentration of sample required to chelate 50% of the metallic ions. µM CE: µM of catechin equivalents (CE). The enzymatic reaction contained polyphenol compounds (PCs) at 1.5 mM concentration, 2.5 U/mL of peroxidase and 0.05% hydrogen peroxide (H_2_O_2_). In the control reaction, peroxidase was substituted by phosphate buffer solution. EDTA = ethylendiaminetetraacetic acid. Letters ^a–g^ represents the significant differences estimated by ANOVA followed by the Tukey test.

## References

[1] Miksits M., Wlcek K., Svoboda M., Kunert O., Haslinger E., Thalhammer T., Szekeres T., Jäger W. (2009). Antitumor Activity of Resveratrol and its Sulfated Metabolites Against Human Breast Cancer Cells. Planta Med..

[2] Philips B.J., Coyle C.H., Morrisroe S.N., Chancellor M.B., Yoshimura N. (2009). Induction of Apoptosis in Human Bladder Cancer Cells by Green Tea Catechins. Biomed. Res..

[3] Yang S., Ma J., Xiao J., Lv X., Li X., Yang H., Liu Y., Feng S., Zhang Y. (2012). Arctigenin Anti-Tumor Activity in Bladder Cancer T24 Cell Line Through Induction of Cell-Cycle Arrest and Apoptosis. Anat. Rec..

[4] Abdulkhaleq L.A., Assi M.A., Noor M.H.M., Abdullah R., Saad M.Z., Taufiq-Yap Y.H. (2017). Therapeutic Uses of Epicatechin in Diabetes and Cancer. Vet. World.

[5] Hajiaghaalipour F., Kanthimathi M.S., Sanusi J., Rajarajeswaran J. (2015). White Tea (Camellia Sinensis) Inhibits Proliferation of the Colon Cancer Cell Line, HT-29, Activates Caspases and Protects DNA of Normal Cells Against Oxidative Damage. Food Chem..

[6] Avtanski D., Poretsky L. (2018). Phyto-Polyphenols as Potential Inhibitors of Breast Cancer Metastasis. Mol. Med..

[7] Gajera H.P., Gevariya S.N., Hirpara D.G., Patel S.V., Golakiya B.A. (2017). Antidiabetic and Antioxidant Functionality Associated with Phenolic Constituents from Fruit Parts of Indigenous Black Jamun (Syzygium Cumini L.) Landraces. J. Food Sci. Technol..

[8] Fukui M., Choi H.J., Zhu B.T. (2010). Mechanism for the Protective Effect of Resveratrol Against Oxidative Stress-Induced Neuronal Death. Free Radic. Biol. Med..

[9] Xueyan R., Jia Y., Xuefeng Y., Lidan T., Qingjun K. (2018). Isolation and Purification of Five Phenolic Compounds from the Xinjiang Wine Grape (Vitis Vinifera) and Determination of their Antioxidant Mechanism at Cellular Level. Eur. Food Res. Technol..

[10] Nichols J.A., Katiyar S.K. (2010). Skin Photoprotection by Natural Polyphenols: Anti-Inflammatory, Antioxidant and DNA Repair Mechanisms. Arch. Dermatol. Res..

[11] Prasain J.K., Jones K., Moore R., Barnes S., Leahy M., Roderick R., Juliana M.M., Grubbs C.J. (2008). Effect of Cranberry Juice Concentrate on Chemically-Induced Urinary Bladder Cancers. Oncol. Rep..

[12] Shingai Y., Fujimoto A., Nakamura M., Masuda T. (2011). Structure and Function of the Oxidation products of Polyphenols and Identification of Potent Lipoxygenase inhIbitors from Fe-Catalyzed Oxidation of Resveratrol. J. Agric. Food Chem..

[13] Jeon S.Y., Oh S., Kim E., Imm J.Y. (2013). α-Glucosidase Inhibiton and Antiglycation Activity of Laccase-Catalyzed Catechin Polymers. J. Agric. Food Chem..

[14] American Cancer Society Cancer Facts and Figures. https://www.cancer.org/content/dam/cancer-org/research/cancer-facts-and-statistics/annual-cancer-facts-and-figures/2019/cancer-facts-and-figures-2019.pdf.

[15] Stocco B., Toledo K., Salvador M., Paulo M., Koyama N., Torqueti Toloi M.R. (2012). Dose-Dependent Effect of Resveratrol on Bladder Cancer Cells: Chemoprevention and Oxidative Stress. Maturitas.

[16] López-Serrano M., Ros Barceló A. (2002). Comparative Study of the Products of the Peroxidase-Catalyzed and the Polyphenoloxidase-Catalyzed (+)-Catechin Oxidation. Their Possible Implications in Strawberry (Fragaria X Ananassa) Browning Reactions. J. Agric. Food Chem..

[17] Blainski A., Lopes G.C., De Mello J.C.P. (2013). Application and Analysis of the Folin Ciocalteu Method for the Determination of the Total Phenolic Content from Limonium Brasiliense L. Molecules.

[18] Dávalos A., Gómez-Cordovés C., Bartolomé B. (2004). Extending Applicability of the Oxygen Radical Absorbance Capacity (ORAC-Fluorescein) Assay. J. Agric. Food Chem..

[19] Sánchez-Vioque R., Polissiou M., Astraka K., De Los Mozos-Pascual M., Tarantilis P., Herraiz-Peñalver D., Santana-Méridas O. (2013). Polyphenol Composition and Antioxidant and Metal Chelating Activities of the Solid Residues from the Essential Oil Industry. Ind. Crop. Prod..

[20] Bahuguna A., Khan I., Bajpai V.K., Kang S.C. (2017). MTT Assay to Evaluate the Cytotoxic Potential of a Drug. Bangladesh J. Pharmacol..

[21] Munoz-Munoz J.L., García-Molina F., Molina-Alarcón M., Tudela J., García-Cánovas F., Rodríguez-López J.N. (2008). Kinetic Characterization of the Enzymatic and Chemical Oxidation of the Catechins in Green Tea. J. Agric. Food Chem..

[22] Hosny M., Rosazza J.P.N. (2002). Novel Oxidations of (+)-Catechin by Horseradish Peroxidase and Laccase. J. Agric. Food Chem..

[23] Vermerris W., Nicholson R.L., Vermerris W., Nicholson R.L. (2006). Chemical properties of phenolic compounds. Phenolic Compound Biochemistry.

[24] Reihmann M., Ritter H., Kobayashi S., Ritter H., Kaplan D. (2005). Synthesis of phenol polymers using peroxidases. Enzyme-Catalyzed Synthesis of Polymers.

[25] Niki E. (2010). Assessment of Antioxidant Capacity In Vitro and In Vivo. Free Radic. Biol. Med..

[26] Silva M.M., Santos M.R., Caroҫo G., Rocha R., Justino A., Mira L. (2002). Structure-Antioxidantactivity Relationships of Flavonoids: A Re-Examination. Free Radic. Res..

[27] Chebil L., Rhouma G.B., Chekir-Ghedira L., Ghoul M., Ekinci D. (2015). Enzymatic polymerization of rutin and esculin and evaluation of the antioxidant capacity of polyrutin and polyesculin. Biotechnology.

[28] Nicotra S., Cramarossa M.R., Mucci A., Pagnoni U.M., Riva S., Forti L. (2004). Biotransformation of Resveratrol: Synthesis of Trans-Dehydrodimers Catalyzed by Laccases from Myceliophtora Thermophyla and from Trametes Pubescens. Tetrahedron.

[29] Kurisawa M., Chung J.E., Kim Y.J., Uyama H., Kobayashi S. (2003). Amplification of Antioxidant Activity and Xanthine Oxidase Inhibition of Catechin by Enzymatic Polymerization. Biomacromolecules.

[30] Racicot K., Favreau N., Fossey S., Grella A.R., Ndou T., Bruno F.F. (2012). Antioxidant Potency of Highly Purified Polyepicatechin Fractions. Food Chem..

[31] Yu B.B., Han X.Z., Lou H.X. (2007). Oligomers of Resveratrol and Ferulic acid Prepared by Peroxidase-Catalyzed Oxidation and their Protective Effects on Cardiac Injury. J. Agric. Food Chem..

[32] Perron N.R., Brumaghim J.L. (2009). A Review of the Antioxidant Mechanisms of Polyphenol Compounds Related to Iron Binding. Cell Biochem. Biophys..

[33] Letelier M.E., Sánchez-Jofré S., Peredo-Silva L., Cortés-Troncoso J., Aracena-Parks P. (2010). Mechanisms Underlying Iron and Copper Ions Toxicity in Biological Systems: Pro-Oxidant Activity and Protein-Binding Effects. Chem. Biol. Interact..

[34] Karuppagounder S.S., Pinto J.T., Xu H., Chen H.L., Beal M.F., Gibson G.E. (2009). Dietary Supplementation with Resveratrol Reduces Plaque Pathology in a Transgenic Model of Alzheimer’s Disease. Neurochem. Int..

[35] Weinreb O., Amit T., Mandel S., Youdim M.B.H. (2009). Neuroprotective Molecular Mechanisms of (-)-Epigallocatechin-3-Gallate: A Reflective Outcome of its Antioxidant, Iron Chelating and Neuritogenic Properties. Genes Nutr..

[36] Peguero J.G., Arenas I., Lamas G.A. (2014). Chelation Therapy and Cardiovascular Disease: Connecting Scientific Silos to Benefit Cardiac Patients. Trends Cardiovas. Med..

[37] Heath J.L., Weiss J.M., Lavau C.P., Wechsler D.S. (2013). Iron Deprivation in Cancer—Potential Therapeutic Implications. Nutrients.

[38] Nagarajan S., Nagarajan R., Braunhut S.J., Bruno F., McIntosh D., Samuelson L., Kumar J. (2008). Biocatalytically Oligomerized Epicatechin with Potent and Specific Anti-Proliferative Activity for Human Breast Cancer Cells. Molecules.

[39] Ito T., Akao Y., Yi H., Ohguchi K., Matsumoto K., Tanaka T., Iinuma M., Nozawa Y. (2003). Antitumor Effect of Resveratrol Oligomers Against Human Cancer Cell Lines and the Molecular Mechanism of Apoptosis Induced by Vaticanol C. Carcinogenesis.

[40] Fernández-Rojas B., Medina-Campos O.N., Hernández-Pando R., Negrette-Guzmán M., Huerta-Yepez S., Pedraza-Chaverri J. (2014). C-Phycocyanin Prevents Cisplatin-Induced Nephrotoxicity through Inhibition of Oxidative Stress. Food Funct..

[41] Xue Y.Q., Di J.M., Luo Y., Cheng K.J., Wei X., Shi Z. (2014). Resveratrol Oligomers for the Prevention and Treatment of Cancers. Oxid. Med. Cell. Longev..

[42] Qin J., Wang Y., Bai Y., Yang K., Mao Q., Lin Y., Kong D., Zheng X., Xie L. (2012). A Component of Green Tea, (-)-Epigallocatechin-3-Gallate, Promotes Apoptosis in T24 Human Bladder Cancer Cells via Modulation of the PI3K/Akt Pathway and Bcl-2 Family Proteins. Mol. Med. Rep..

